# Comparison of respiratory pathogen colonization and antimicrobial susceptibility in people with cystic fibrosis bronchiectasis versus non-cystic fibrosis bronchiectasis: a protocol for a systematic review

**DOI:** 10.1186/s13643-020-01557-6

**Published:** 2021-01-04

**Authors:** Salony Verma, Joseph L. Mathew, Pallab Ray

**Affiliations:** 1 Department of Medical Microbiology, Post Graduate Institute of Medical Education and Research, Chandigarh, 160012 India; 2 Department of Pediatrics, Post Graduate Institute of Medical Education and Research, Chandigarh, 160012 India

**Keywords:** Bronchiectasis, Cystic fibrosis bronchiectasis, Non-CF bronchiectasis, Antimicrobial susceptibility, Bacterial colonization, Antibiotic, Resistance, Susceptibility

## Abstract

**Background:**

Both cystic fibrosis (CF) and non-cystic fibrosis bronchiectasis are characterized by permanent bronchial dilation, impaired mucociliary clearance, and development of chronic colonization and infection. Although the major airway microbiota in both CF and non-CF bronchiectasis may be similar, there are some differences in clinical and microbiologic features. There may also be differences in antibiotic susceptibility patterns between the CF and non-CF populations. Therefore, analysis and comparison of the microbiota and antibiotic susceptibility pattern in CF bronchiectasis versus non-CF bronchiectasis would help to improve the management of both conditions.

**Methods:**

Two authors will independently search the electronic databases PubMed, EMBASE, the Cochrane Library, and LIVIVO, for studies reporting bacterial colonization of the respiratory tract in adults and children diagnosed with bronchiectasis in either CF or non-CF. We will include studies examining any respiratory tract specimen, using conventional bacterial culture or other specialized techniques such as molecular methods. We will also examine the antimicrobial susceptibility patterns in people with CF bronchiectasis versus non-CF bronchiectasis. The authors will independently assess the risk of bias in each included study using the Newcastle Ottawa Scale (NOS). We will present the data with descriptive statistics and provide pooled estimates of outcomes, wherever it is feasible to perform meta-analysis. Heterogeneity in studies will be explored by visual inspection of forest plots as well as using the Higgins and Thompson *I*^2^ method. We will contact the corresponding authors of studies where data is/are missing and try to obtain the missing data. We will undertake sensitivity analysis to explore the impact of study quality and subgroup analysis based on pre-set criteria. We will prepare a summary of findings’ table and assess the confidence in the evidence using the GRADE methodology.

**Discussion:**

To date, there are no locally applicable evidence-based guidelines for antimicrobial treatment of non-CF bronchiectasis patients. In general, treatment is based on extrapolation of evidence in people with CF bronchiectasis. An insight into the microbiota and antimicrobial susceptibility patterns in the two conditions would facilitate appropriate rather than empiric antimicrobial therapy and hopefully reduce the burden of antimicrobial resistance created by rampant usage of antibiotics.

**Systematic review registration:**

The protocol has been registered in PROSPERO on July 26, 2020 (PROSPERO registration number: CRD42020193859).

**Supplementary Information:**

The online version contains supplementary material available at 10.1186/s13643-020-01557-6.

## Background

Cystic fibrosis (CF) is an autosomal recessive disorder caused by mutations in cystic fibrosis transmembrane conductance regulator (CFTR) gene. This gene regulates the activity of sodium and chloride channels across the epithelial cells, thereby facilitating appropriate hydration of mucins and effective muco-ciliary clearance in various organs [1]. Impaired secretion of chloride and bicarbonate ions due to CFTR mutations leads to the formation of thick mucus, which is difficult to clear [2]. This predisposes CF patients to pulmonary bacterial infections caused by *Staphylococcus aureus*, *Pseudomonas aeruginosa*, *Haemophilus influenzae*, or *Burkholderia cepacia* complex (Bcc) [3]. The inflammatory response to recurrent infections eventually leads to bronchiectasis, characterized by permanent bronchial dilation. This causes bacterial adherence, increased bacterial load, and the development of chronic infection. The bacteria gradually adapt to these conditions by forming biofilms, protecting them from phagocytosis as well as penetration of antibiotics [4].

Besides CF, bronchiectasis is associated with various other conditions such as immunodeficiency disorders, autoimmune diseases, ciliary abnormalities, connective tissue diseases, airway injury, malignancy, inflammatory bowel disease, alpha-1 antitrypsin deficiency, or hypersensitivity (allergic bronchopulmonary aspergillosis). These are collectively termed as non-CF bronchiectasis [5].

There are many similarities between CF and non-CF bronchiectasis. Both are associated with severe airway inflammation, mucus obstruction, reduced lung function, progression over time, and frequent pulmonary exacerbations [6, 7]. However, there are also many differences between the two conditions.

Various biological specimens have been analyzed for identifying bacterial colonization patterns in people with bronchiectasis. Sputum is the preferred specimen for culturing bacteria and is also tested for acid-fast bacilli in non-CF bronchiectasis [5]. Bronchoalveolar lavage (BAL) is reserved for patients who are unable to produce sputum or when sputum does not yield organisms. Epithelial lining fluid (ELF) is another respiratory specimen used for microbiologic analysis.

The major microbiota colonizing the airways are similar in both CF and non-CF bronchiectasis. *Staphylococcus aureus*, *Pseudomonas aeruginosa*, *Burkholderia cepacia* complex, *Haemophilus influenzae*, *Stenotrophomonas maltophilia*, and *Achromobacter xylosoxidans* are commonly associated with CF bronchiectasis [8] while *Haemophilus influenzae*, *Pseudomonas aeruginosa*, *Moraxella catarrhalis*, *or non-tuburculous Mycobacteria* (NTM) are the predominant bacterial species associated with non-CF bronchiectasis [5, 9]. Gram-positive bacteria including *Streptococcus pneumoniae* and *Staphylococcus aureus* are rarely associated with non-CF bronchiectasis unlike CF bronchiectasis [10]. Further, the core microbiota in both the conditions are similar in childhood but diverge by adulthood [6, 11].

Antibiotics are the mainstay of treatment of bronchiectasis in both CF and non-CF patients. The choice of antibiotics is based on an understanding of the predominant respiratory tract colonizers in individual patients, as well as local antimicrobial susceptibility testing (AST) patterns. The appropriate use of antibiotics is associated with improved pulmonary function and survival [12].

AST may predict the success or failure of specific antibiotics, by sorting out the resistant bacteria from the susceptible ones on the basis of Minimal Inhibitory Concentration (MIC) breakpoints. These are determined by breakpoint committees such as the European Committee on Antimicrobial Susceptibility Testing (EUCAST) or the Clinical and Laboratory Standards Institute (CLSI). However, the epidemiological cut-off is determined using the susceptibility data from the wild-type population and does not take into consideration any mutant strains [13], which are commonly encountered in the mucus-obstructed airways of CF patients. Therefore, people with CF bronchiectasis often receive antibiotics in higher doses and for longer duration, compared to non-CF bronchiectasis [14]. So clinicians cannot rely only on such data for prescribing empirical therapy to the CF patients. There are several other factors, responsible for differences in antibiotic susceptibility patterns between CF and non-CF populations. Bacteria in CF airways respond to the deficient oxygen or nutrient conditions by slowing down their growth rate or by altering their metabolism [4], which fosters resistance to several antibiotics. Therefore, antibiotics acting on the cell-wall may not be effective in eradicating bacteria that are not actively dividing or are growing slowly. Many bacteria form biofilms, which make them impervious to antibiotics [15]. In addition, different colonial types of bacteria such as small colony variants (SCV) are observed in the respiratory specimens of CF patients [16, 17], which are often missed in routine laboratory testing. A single specimen from CF patients may contain mixed populations of a single organism with different antibiotic susceptibility profile [18].

Therefore, a detailed comparison of respiratory pathogen colonization in people with CF bronchiectasis and non-CF bronchiectasis, and the antimicrobial susceptibility patterns in them, could improve management of both conditions.

### Aim and objectives of the systematic review

This systematic review aims to compare the microbiota and antimicrobial susceptibility profile in people with CF bronchiectasis versus non-CF bronchiectasis. We propose to undertake a systematic review of literature to address the following research questions:

1. What are the bacteria colonizing the respiratory tract in people with cystic fibrosis bronchiectasis, compared to non-cystic fibrosis bronchiectasis?
2. How does the antibiotic susceptibility profile of specific bacteria, differ between people with CF bronchiectasis versus non-CF bronchiectasis?

## Methods/design

The protocol of this systematic review has been registered in the PROSPERO database, the International prospective register of systematic reviews on July 26, 2020 (CRD42020193859). This protocol is in accordance with the Preferred Reporting Item for Systematic Review and Meta-analysis (PRISMA-P) guidelines (Additional file 1) [19].

### Types of studies

The data required for this review could be available in observational studies including cohort studies or case-control studies (case arm), one or other arm of controlled clinical trials (randomized or non-randomized), or case series.

### Types of participants

Adults and children diagnosed with CF bronchiectasis or non-CF bronchiectasis.

### Inclusion criteria

We will include studies reporting bacterial colonization of the respiratory tract determined by examination of any respiratory tract specimen, by conventional bacterial culture or molecular techniques, and/or antimicrobial susceptibility testing by any method.

### Exclusion criteria

We will exclude the following studies:

1. Those which include people with CF or non-CF conditions, but without bronchiectasis.
2. Those in which data of patients with and without bronchiectasis cannot be distinguished.
3. Those in which the underlying cause(s) of bronchiectasis cannot be distinguished as CF or non-CF.
4. Those wherein multiple clinical conditions were studied, but it is not possible to obtain or analyze data for CF or non-CF bronchiectasis.
5. Those reporting microbiology data wherein the underlying clinical condition(s) are not specified.
6. Those in which non-standard culture methods were used to identify organisms.
7. Case series with less than 10 participants.
8. Studies conducted in animals or animal models, or studies wherein already identified organisms were evaluated further for genotypic or phenotypic characteristics.

### Comparisons considered in this review

1. Clinical or microbiological studies of any design (observational, controlled clinical trials, or case series), reporting bacterial colonization of the respiratory tract (from any type of biological specimen) in CF and non-CF bronchiectasis, and/or antimicrobial susceptibility in both types of patients. Such studies will be considered direct comparisons.
2. Clinical or microbiological studies of any design (observational, controlled clinical trials, or case series), reporting bacterial colonization of the respiratory tract, and/or antimicrobial susceptibility in either CF or non-CF patients, within a 3-year period. Such studies will be considered indirect comparisons.
3. Clinical or microbiological studies of any design (observational, controlled clinical trials, or case series), reporting bacterial colonization of the respiratory tract, and/or antimicrobial susceptibility in either CF or non-CF patients, within any time period, if they are from the same institution. Such studies will also be considered indirect comparisons.

### Types of outcome measures

1. Bacteria identified in the respiratory tract in people with CF bronchiectasis versus non-CF bronchiectasis.
2. Relative proportion of various bacterial species identified in the respiratory tract in people with CF bronchiectasis versus non-CF bronchiectasis.
3. Number of bacterial species identified per person with CF bronchiectasis versus non-CF bronchiectasis.
4. Bacteria identified in people with non-CF bronchiectasis, with a known cause versus unknown cause(s).
5. Proportion of specific bacterial species susceptible to specific antimicrobial agents.
6. Proportion of specific bacterial species resistant to specific antimicrobial agents.
7. Proportion of specific bacterial species with intermediate susceptibility to specific antimicrobial agents.
8. Time-trend of antimicrobial sensitivity patterns in 10-year epochs.

### Search methods for identification of studies

**Fig. 1:**
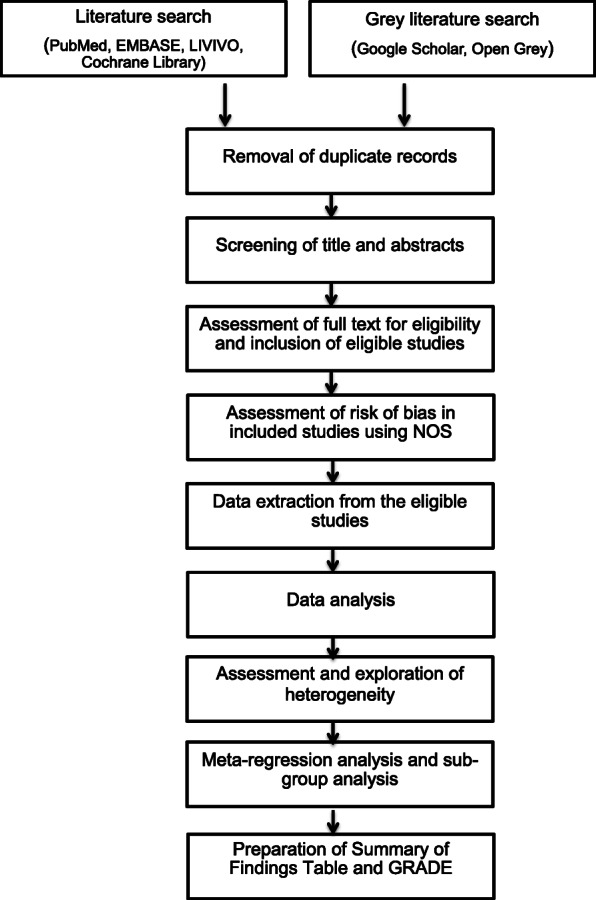
Flowchart of the systematic review

Two authors will independently search the electronic databases PubMed, EMBASE, the Cochrane Library, and LIVIVO. Grey literature will be searched using the search engines, Open Grey and Google Scholar. Figure 1 presents a flowchart of the systematic review. MeSH terms for keywords will be used as follows:

1. Bronchiectasis AND Cystic fibrosis AND non-cystic fibrosis AND (antibiotic OR antimicrobial) AND (susceptibility OR sensitivity OR resistance)
2. Cystic fibrosis AND bronchiectasis AND (antibiotic OR antimicrobial) AND (susceptibility OR sensitivity OR resistance)
3. Non-cystic fibrosis AND bronchiectasis AND (antibiotic OR antimicrobial) AND (susceptibility OR sensitivity OR resistance)
4. Pulmonary exacerbation AND (antibiotic OR antimicrobial) AND (susceptibility OR sensitivity OR resistance)
5. Bronchiectasis AND Cystic fibrosis AND non-cystic fibrosis AND (microbiota OR pathogens OR colonizers OR bacteria OR microbiology)
6. Cystic fibrosis AND bronchiectasis AND (microbiota OR pathogens OR colonizers OR bacteria OR microbiology)
7. Non-cystic fibrosis AND bronchiectasis AND (microbiota OR pathogens OR colonizers OR bacteria OR microbiology)

### Data collection and analysis

Studies will be screened by examining titles and abstracts. Those identified as potentially relevant will be retrieved and full text examined. Each full-text article will be evaluated for eligibility to be included in the review. There will be no language restriction. Manual search of the reference lists of the included studies will also be performed. We will inform the date last searched for each of the databases. Disagreements/discrepancies will be resolved by discussion and if necessary, arbitration by the third author.

### Data extraction and management

Data will be independently extracted by two reviewers into a special data extraction form.

From each included study, the following information will be extracted: Identification data (author, year), study design, institution, country or countries, time-period of study, whether clinical or microbiological analysis, inclusion criteria of patients (CF and non-CF bronchiectasis), age of patients, clinical state of patients (stable, acute exacerbation, surveillance culture, etc.), underlying clinical condition, duration of illness if known, presence of co-morbidity, specimen tested, specimen collection method, whether already on antibiotics, micro-organisms identified, bacteriological method used for identification (culture, molecular methods, etc.), bacterial quantification, biofilm formation, antimicrobial susceptibility profile, method of testing antimicrobial susceptibility, definition of sensitivity, and clinical/bacteriological outcome (Tables 1 and 2).

**Table 1:** Respiratory pathogen colonization in people with cystic fibrosis bronchiectasis versus non-cystic fibrosis bronchiectasis

**Data extracted by**		
**Reference**	Author & year	
	Citation	
	Country/countries	
	Institution/s	
**Study design**		
**Clinical/microbiology study**		
• Observational study		
• Clinical trial		
• Case series with > 10 cases		
• Case series with < 9 case		
**Time period**		
**Participants**	• CF bronchiectasis	
	• Non-CF bronchiectasis	
	• Mixed	
	• Other	
**Study group (CF bronchiectasis)**	No.	
	Age	
	• Adults > 18 years	
	• Children < 18 years	
	• Mixed (not possible to separate)	
	Sex	
	**Respiratory pathogens**	**No. (%)**
	*Staphylococcus aureus (MSSA)*	
	*Staphylococcus aureus (MRSA)*	
	*Pseudomonas aeruginosa*	
	*Burkholderia 20 epacian* complex	
	*Haemophilus influenzae*	
	*Stenotrophomonas maltophilia*	
	*Achromobacter xylosoxidans*	
	NTM	
	*Streptococcus*	
	*Veillonella*	
	*Prevotella*	
	*Others*	
	Inclusion/Exclusion criteria	
	Sample type (sputum/BAL/ELF)	
	Upper respiratory specimen	
	• Nasal specimen	
	• Nasopharyngeal specimen	
	• Oropharyngeal specimen	
	• Other	
	Lower respiratory specimen	
	• Sputum	
	• Induced sputum	
	• BAL	
	• Lung aspirate	
	• Other	
	**Antibiotic therapy**	
	• Already on antibiotic(s)	
	• Not on antibiotic(s)	
	• Unclear	
	**Method of identifying species**	
	• Culture	
	• PCR	
	• Other	
	**Antimicrobial resistance**	
	Yes/No	
**Comparison group (non-CF bronchiectasis)**	Cause of non-CF bronchiectasis/diagnosis	
	No.	
	Age	
	Adults > 18 years	
	Children < 18 years	
	Mixed (not possible to separate)	
	Sex	
	**Respiratory pathogens**	**No. (%)**
	*Haemophilus influenzae*	
	*Pseudomonas aeruginosa*	
	*Moraxella catarrhalis*	
	NTM	
	*Staphylococcus aureus*	
	*Streptococcus*	
	*Veillonella*	
	*Prevotella*	
	Others	
	Inclusion/exclusion criteria	
	Sample type (sputum/BAL/ELF)	
	Upper respiratory specimen	
	• Nasal specimen	
	• Nasopharyngeal specimen	
	• Oropharyngeal specimen	
	• Other	
	Lower respiratory specimen	
	• Sputum	
	• Induced sputum	
	• BAL	
	• Lung aspirate	
	• Other	
	**Antibiotic therapy**	
	• Already on antibiotic(s)	
	• Not on antibiotic(s)	
	• Unclear	
	**Method of identifying species**	
	• Culture	
	• PCR	
	• Other	
	**Clinical state**	
	• Acute pulmonary exacerbation	
	• Surveillance culture	
	**Antimicrobial resistance**	
	Yes/No	
**Remarks**

**Table 2:** Antibiotic susceptibility profile in people with cystic fibrosis versus non cystic fibrosis bronchiectasis

Data extracted by										
Reference	Author & year									
	Citation									
	Country/countries									
	Institution/s									
Study design	Clinical/microbiology study									
	Observational study									
	Clinical trial									
	Case series with > 10 cases									
	Case series with < 9 case									
Time period										
Participants	• CF bronchiectasis									
	• Non CF bronchiectasis									
	• Mixed									
	• Other									
**Study group (CF bronchiectasis)**	No.									
	Age									
	• Adults > 18 years									
	• Children < 18 years									
	• Mixed (not possible to separate)									
	Sex									
	AST method									
	• AST definition									
	• Ability to detect mutants									
	• Advantages									
	• Disadvantages									
	**Antibiotic susceptibility (S/I/R; MIC)**									
**Microorganism**	Tobramycin		Azythromycin	Carbenicillin	Ceftazidime	Gentamycin	Chloramphenicol	Ciprofloxacin	Colistin	Minocycline
*Staphylococcus aureus* (MSSA)										
*Staphylococcus aureus* (MRSA)										
*Pseudomonas aeruginosa*										
*Burkholderia cepacia* complex										
*Haemophilus influenzae*										
*Stenotrophomonas maltophilia*										
*Achromobacter xylosoxidans*										
NTM										
*Streptococcus*										
*Veillonella*										
Others										
Inclusion/Exclusion criteria										
**Upper respiratory specimen**										
Nasal specimen										
Nasopharyngeal specimen										
Oropharyngeal specimen										
Other										
**Lower respiratory specimen**										
Sputum										
Induced sputum										
BAL										
Lung aspirate										
Other										
**Antibiotic therapy**										
Already on antibiotic(s)										
Not on antibiotic(s)										
Unclear										
**Method of identifying species**										
Culture										
PCR										
Other										
Biofilm detection										
Antimicrobial resistance (Yes/No)										
Remarks										
**Comparison group (non-CF bronchiectasis)**	No.									
	Age									
	• Adults > 18 years									
	• Children < 18 years									
	• Mixed (not possible to separate)									
	Sex									
	AST method									
	• AST definition									
	• Ability to detect mutants									
	• Advantages									
	• Disadvantages									
	**Antibiotic susceptibility (S/I/R; MIC)**									
**Microorganism**	Piperacillin-tazobactam	Amoxycillin	Clarithromycin	Ciprofloxacin	Ceftazidime	Cefuroxime	Cefotaxime	Flucloxacillin	Tobramycin	Amoxicillin-clavulanate
*Haemophilus influenzae*										
*Pseudomonas aeruginosa*										
*Moraxella catarrhalis*										
NTM										
*Staphylococcus aureus*										
*Streptococcus*										
*Veillonella*										
*Prevotella*										
Others										
Inclusion/Exclusion criteria										
**Upper respiratory specimen**										
Nasal specimen										
Nasopharyngeal specimen										
Oropharyngeal specimen										
Other										
**Lower respiratory specimen**										
Sputum										
Induced sputum										
BAL										
Lung aspirate										
Other										
**Antibiotic therapy**										
Already on antibiotic(s)										
Not on antibiotic(s)										
Unclear										
**Method of identifying species**										
Culture										
PCR										
Other										
Biofilm detection										
Antimicrobial resistance (Yes/No)										
Remarks										

### Assessment of risk of bias in included studies

Two authors will independently assess the risk of bias in each included study using the Newcastle Ottawa Scale (NOS) [20]. The NOS contains eight items, categorized into three broad perspectives: selection of the study groups, comparability of the groups, and ascertainment of either the exposure or outcome of interest for case-control or cohort studies, respectively. For each item, a series of response options is provided. A star system is used to allow a semi-quantitative assessment of study quality. A study can be awarded a maximum of one star for each numbered item within the selection and exposure categories. A maximum of two stars can be given for comparability. High-quality studies will be defined as those having an NOS score > 6, from a maximum of 9 points [21].

### Statistical analysis

We will present the data with descriptive statistics and provide pooled estimates of outcome parameters, wherever it is feasible to perform meta-analysis. Pooled estimates will be presented with a 95% confidence interval. The default analysis will be with a random effects model.

### Dealing with missing data

We will contact the corresponding authors of studies where data is/are missing and try to obtain the missing data. If this fails, we will try and impute data where possible. If that is not feasible, we will state as such.

### Assessment of heterogeneity

Heterogeneity in studies will be explored by visual inspection of forest plot as well as using the Higgins and Thompson *I*^2^ method [22]. The *I*^2^ heterogeneity will be categorized as follows: 0–50% low, 50–75% moderate, and > 75% considerable heterogeneity. Where *I*^2^ is greater than 50%, we will try to identify possible explanations using subgroup analysis and meta-regression analysis based on the most important characteristics of the studies.

### Assessment of reporting biases

Wherever possible, we will obtain the original trial protocols for comparison with the published papers to ensure that all outcomes were reported. If it is not possible to obtain the trial protocols, we will scrutinize the “Methods” section of the published paper(s) to ensure full reporting of all measured variables. If negative data were not fully reported, we will contact the primary investigators for these data. We will explore reporting bias using a funnel plot. We will also assess publication bias by looking for evidence of conference presentations not followed by subsequent journal publications.

### Sensitivity analysis

In order to assess whether the results are substantially influenced by the presence of any individual study, we will perform a sensitivity analysis by systematically removing studies with a high risk of bias and recalculating the results.

### Subgroup analysis and investigation of heterogeneity

We will analyze results separately by the following characteristics:

1. Age group of patients: Children (< 18 years) versus adults (> 18 years).
2. Antibiotic therapy: Already on antibiotics versus not on antibiotics.
3. Clinical state: During acute exacerbations versus stable clinical state, i.e., surveillance culture.
4. Type of respiratory specimen: Upper respiratory tract specimen versus lower respiratory tract specimen.
5. Method used for identification of bacterial species: Culture versus molecular methods or other non-culture methods.

### Summary of findings table and GRADE

We will present two summaries of findings’ tables; one comparing the bacteria identified in respiratory specimens in those with CF bronchiectasis versus non-CF bronchiectasis (Table 1), and the other comparing antimicrobial susceptibility patterns of individual bacterial species in CF bronchiectasis versus non-CF bronchiectasis (Table 2).

Two reviewers will independently assess the quality of evidence based on five domains, i.e., design (risk of bias), consistency across studies, directness of the evidence, precision of estimates, and presence of publication bias using the Grading of Recommendations Assessment, Development and Evaluation (GRADE) approach [23]. The body of evidence will then be classified as high, moderate, low, and very low.

## Discussion

To date, there are no locally applicable evidence-based guidelines for antimicrobial treatment of non-CF bronchiectasis patients. In general, treatment is based on extrapolation of evidence in CF bronchiectasis [24, 25]. Hence, comparing the bacterial colonization pattern and antibiotic susceptibility profile in CF bronchiectasis versus non-CF bronchiectasis would aid in improved management of both the conditions. Furthermore, the understanding of the microbiota in both CF and non-CF populations would aid in more personalized treatment approaches. Understanding the antimicrobial susceptibility patterns against specific organisms can facilitate appropriate rather than empiric therapy and hopefully reduce the burden of antimicrobial resistance created by rampant usage of antibiotics.

## Supplementary Information

**Additional file 1.** PRISMA-P 2015 Checklist.

## Data Availability

Not applicable.

## Abbreviations

CF: Cystic fibrosis
AST: Antimicrobial susceptibility
NOS: Newcastle Ottawa Scale
MeSH: Medical Subject Headings
PROSPERO: Prospective Register of Systematic Reviews
MIC: Minimum Inhibitory Concentration
BAL: Bronchoalveolar lavage
ELF: Epithelial lining fluid
GRADE: Grading of Recommendations Assessment, Development and Evaluation
PCR: Polymerase chain reaction
